# Lemierre's Syndrome With Multivessel Thrombosis, Right Atrial Extension, and Septic Pulmonary Emboli: An Unusual and Life-Threatening Presentation

**DOI:** 10.7759/cureus.91453

**Published:** 2025-09-02

**Authors:** Usamah Al-Anbagi, Shahd Hammouda, Bassem Al Hariri, Abdulqadir J Nashwan, Hatem M Abusriwil

**Affiliations:** 1 Internal Medicine Department, Hazm Mebaireek General Hospital/Hamad Medical Corporation, Doha, QAT; 2 Medical Education Department, Hamad Medical Corporation, Doha, QAT; 3 Internal Medicine Department, Hamad Medical Corporation, Doha, QAT; 4 Nursing and Midwifery Research Department, Hamad Medical Corporation, Doha, QAT

**Keywords:** case report, internal jugular vein thrombosis, lemierre's syndrome (ls), septic thrombophlebitis, superior vena cava (svc)

## Abstract

Lemierre's syndrome (LS) is a rare but potentially fatal condition characterized by septic thrombophlebitis of the internal jugular vein (IJV), most often secondary to oropharyngeal infection. It is typically associated with *Fusobacterium necrophorum* and may lead to septic emboli, predominantly in the lungs. We report a rare case of a previously healthy 33-year-old man with LS complicated by extensive multivessel thrombosis, including the internal jugular, brachiocephalic, and superior vena cava (SVC) veins, with propagation of thrombus into the right atrium, in addition to septic pulmonary emboli. This constellation of findings is exceedingly rare and underscores the propensity of LS to extend beyond cervical veins into central thoracic and cardiac structures. Despite persistently negative blood cultures, likely due to prior antibiotic exposure, the diagnosis was established through clinical and imaging features. The patient was successfully managed with prolonged intravenous antibiotics, anticoagulation, and a multidisciplinary approach. This case emphasizes the importance of early recognition of atypical and extensive thrombotic complications in LS, as timely intervention can be lifesaving.

## Introduction

Lemierre's syndrome (LS) is a rare but potentially fatal condition characterized by septic thrombophlebitis of the internal jugular vein (IJV), usually following an oropharyngeal infection. First described by André Lemierre in 1936, it was once highly lethal before the advent of antibiotics, with mortality rates approaching 90% [1,2]. Although now uncommon, LS continues to occur, mainly in young, healthy individuals, and can lead to severe complications if diagnosis is delayed [3-7].

The syndrome most often involves *Fusobacterium necrophorum*, an anaerobic Gram-negative bacillus and oropharyngeal commensal, although other bacteria have been implicated [4-6]. Infection spreads from the pharynx to the parapharyngeal space, leading to IJV thrombosis and septic emboli, most commonly to the lungs [1,3,8]. Pulmonary complications include septic emboli, cavitary lesions, and pleural effusions [1,6].

Diagnosis is challenging due to nonspecific symptoms, possible negative blood cultures (particularly after antibiotic exposure), and the rarity of the disease [3,7,8]. Imaging with contrast-enhanced computed tomography (CT) or magnetic resonance imaging (MRI) is key to confirming IJV thrombosis and detecting metastatic lesions [9].

Prolonged targeted antibiotics are the mainstay of treatment, while anticoagulation is considered in extensive thrombosis or septic emboli despite ongoing debate about its benefit [10]. We present a severe, atypical case of LS complicated by superior vena cava (SVC) thrombosis and septic pulmonary emboli, emphasizing the importance of early recognition and multidisciplinary care.

## Case presentation

History

A 33-year-old previously healthy man presented with an eight-day history of right-sided neck swelling that had gradually increased in size. He described mild tenderness in the area but denied any redness or warmth over the skin. Around the same time, he began experiencing episodes of dizziness, especially when lifting heavy objects or leaning forward. He described the dizziness as a spinning sensation that would force him to sit down until it resolved. He had no history of fainting, confusion, or falls. He also denied chest pain, palpitations, or sweating. There were no recent respiratory symptoms, no known sick contacts, and no recent travel.

Physical examination

On arrival, he was febrile at 38.2°C but otherwise hemodynamically stable, with normal oxygen saturation on room air. On examination, his dentition was notably poor, with multiple carious teeth. His throat appeared normal, with no erythema or exudates. There was visible swelling of the right side of the neck, which was non-tender. The jugular venous pressure was elevated but non-pulsatile. Small, non-tender lymph nodes were palpable in both the cervical and inguinal regions. Additionally, there were prominent superficial veins over his chest and abdomen, and his right radial pulse was notably weaker than the left.

Management and follow-up

Initial laboratory results showed a normal white count, slightly prolonged prothrombin time with elevated international normalized ratio (INR), and an elevated C-reactive protein (CRP) (Table 1).

**Table 1:** Laboratory results CRP: C-reactive protein, ALT: alanine aminotransferase, AST: aspartate aminotransferase, PT: prothrombin time, INR: international normalized ratio, APTT: activated partial thromboplastin time, IgG: immunoglobulin G, IgM: immunoglobulin M, ANA: antinuclear antibody, anti-dsDNA: anti-double-stranded DNA, anti-RO52: anti-Ro52 antibody, anti-SS-A: anti-Sjogren's syndrome A antibody, anti-nucleosomes: anti-nucleosome antibody, anti-Sm: anti-Smith antibody, anti-RNP: anti-ribonucleoprotein antibody, anti-histones: anti-histone antibody, anti-PCNA: anti-proliferating cell nuclear antigen antibody, anti-SS-B: anti-Sjogren's syndrome B antibody, anti-ribosomal-P protein: anti-ribosomal P protein antibody, anti-JO1: anti anti-histidyl-tRNA synthetase antibody, anti-AMA-M2: anti-mitochondrial M2 antibody, anti-centromere B: anti-centromere B antibody, anti-PM-Scl: anti-polymyositis scleroderma antibody

Parameters	On admission	On day 6	On day 15	On discharge	Reference values
Total leukocytes	7.8 × 10^3^/uL	6.6 × 10^3^/uL	6.9 × 10^3^/uL	12.2 × 10^3^/uL	6.2 × 10^3^/uL
CRP	136.1 mg/L	90 mg/L	43 mg/L	16 mg/L	0-5 mg/L
Serum urea	2.7 mmol/L	3 mmol/L	2.3 mmol/L	4.2 mmol/L	2.5-7.8 mmol/L
Serum creatinine	67 umol/L	83 umol/L	72 umol/L	84 umol/L	62-106 umol/L
Serum total protein	83 gm/L	72 gm/L	79 gm/L	-	60-80 gm/L
Serum albumin	31 gm/L	24 gm/L	24 gm/L	-	35-50 gm/L
ALT	36 IU/L	68 IU/L	18 IU/L	-	0-41 IU/L
AST	33 IU/L	44 IU/L	14 IU/L	-	0-41 IU/L
Alkaline phosphatase	168 U/L	266 U/L	175 U/L	-	40-129 U/L
PT	15.2 seconds	17 seconds	17 seconds	26.4 seconds	9.4-12.5 seconds
INR	1.4	1.6	1.5	2.4	<1
APTT	6.8 seconds	33.6 seconds	35.1 seconds	43.2 seconds	25.1-36.5 seconds
ANA profile (includes anti-dsDNA, anti-RO52, anti-SS-A, anti-nucleosomes, anti-Sm, anti-RNP, anti-histones, anti-PCNA, anti-SS-B, anti-ribosomal P protein, anti-JO1, anti-AMA-M2, anti-centromere B, anti-PM-Scl antibodies, anti-cardiolipin IgG and IgM, and factor II and V)	All were negative	-	-	-	Negative
Protein C activity	81.5%	-	-	-	70%-140%
Protein S activity	84%	-	-	-	72%-126%
Anti-thrombin activity	88.7%	-	-	-	79.4%-130%

**Figure 1:**
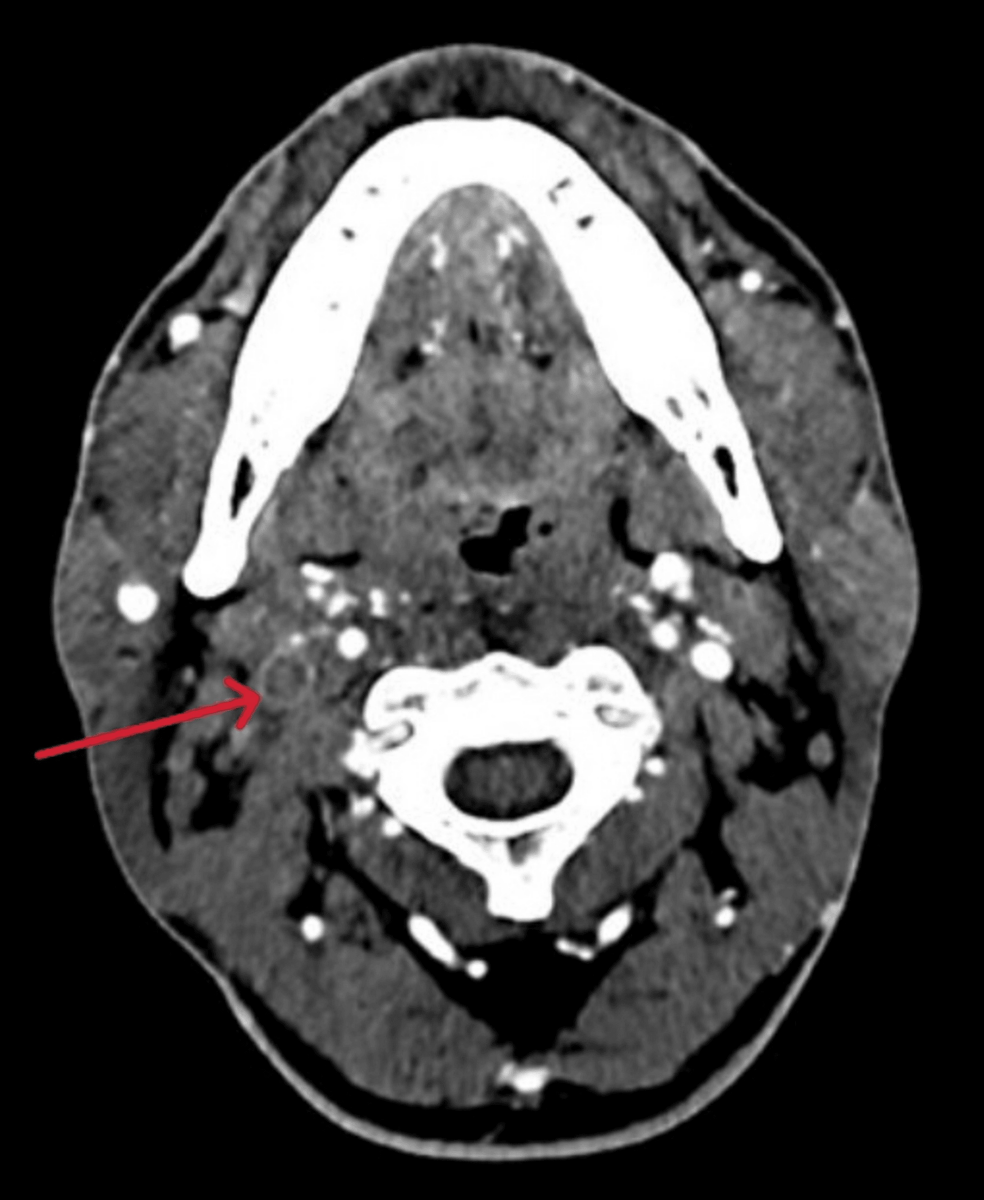
Computed tomography scan (axial view) showing thrombosis of the right internal jugular vein (red arrow)

Given the clinical suspicion of SVC obstruction, he was admitted to the high-dependency unit and started on therapeutic enoxaparin (70 mg twice daily). CT imaging of the neck and thorax confirmed thrombosis in the SVC, right internal jugular, and proximal brachiocephalic veins (Figure 1), along with prevertebral soft tissue edema at the C5-C6 level measuring 15 mm.

**Figure 2:**
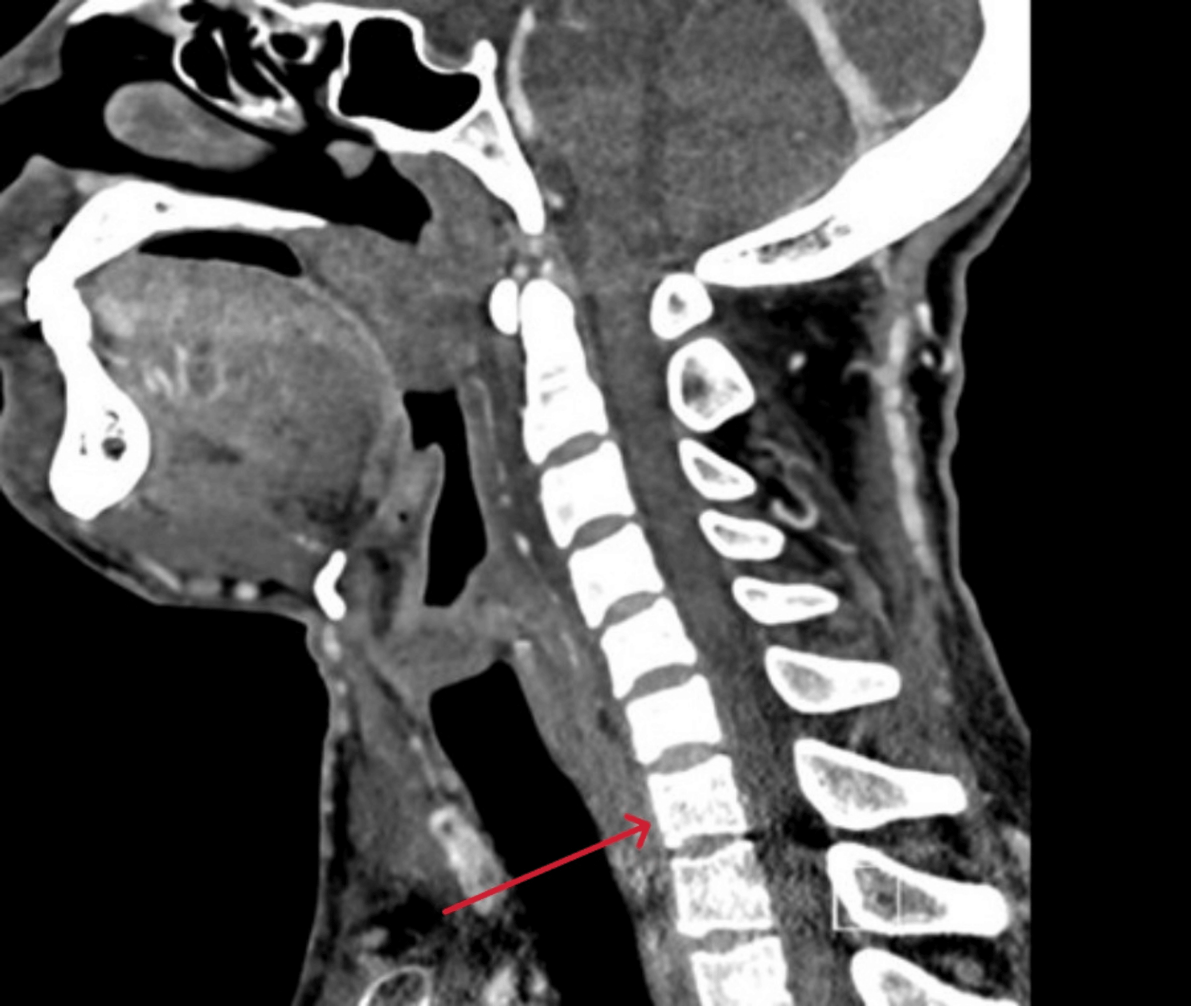
Computed tomography scan (coronal view) showing small prevertebral soft tissue edema at the C5-C6 level (red arrow)

There were also multiple enlarged lymph nodes in the cervical, mediastinal, and axillary regions (Figure 2).

**Figure 3:**
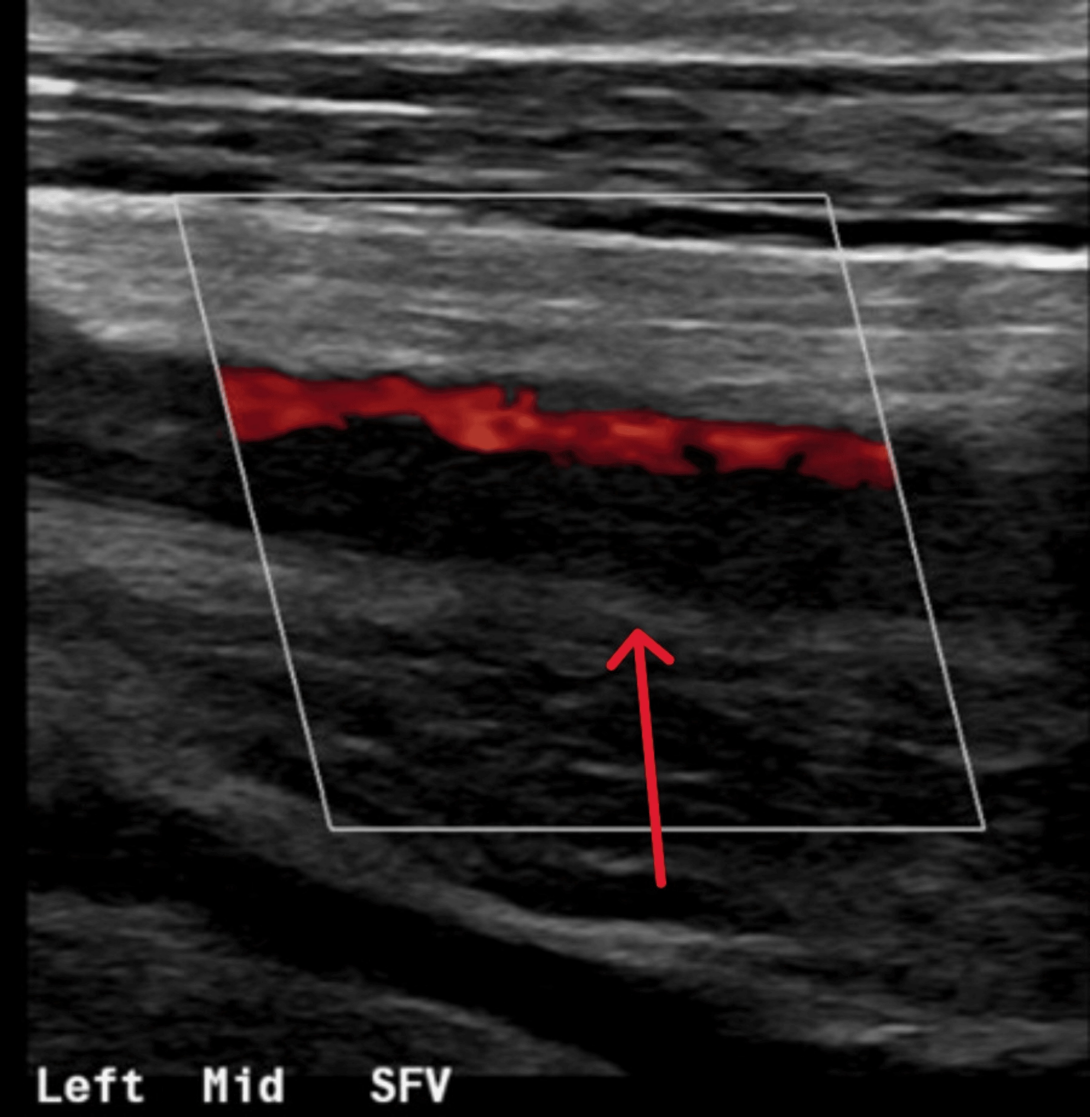
Doppler ultrasound of the left thigh showing deep vein thrombosis of the superficial femoral vein (red arrow)

A follow-up CT three days later showed that the prevertebral collection had decreased in size, from 12.3 mm to 6.4 mm. A CT of the abdomen also revealed thrombi in the left external iliac and femoral veins, and a Doppler ultrasound confirmed extensive deep vein thrombosis (DVT) in the left superficial femoral and popliteal veins (Figure 3).

**Figure 4:**
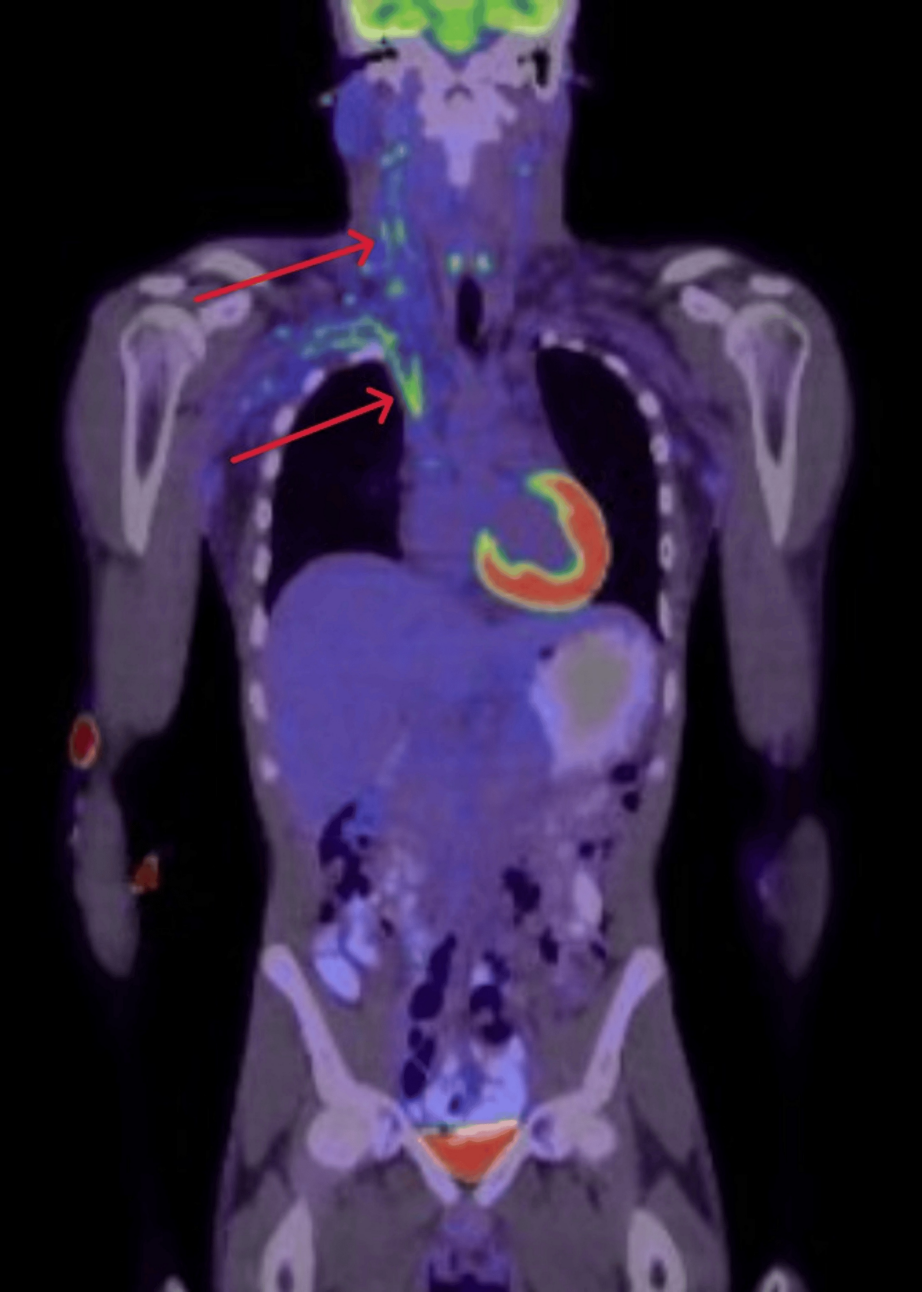
Positron emission tomography scan showing metabolically active thromboses in the neck and thorax, along with lymphadenopathy (red arrows)

He was evaluated by interventional radiology, hematology, and acute care surgery. A biopsy of an axillary lymph node was recommended, but the patient declined. A comprehensive workup was done, including autoimmune and thrombophilia panels, tumor markers, HIV, hepatitis, syphilis testing, paroxysmal nocturnal hemoglobinuria (PNH) screen, serum protein electrophoresis, and multiple sets of blood cultures, all of which were unremarkable (Table 1). A positron emission tomography (PET) scan showed metabolically active thromboses in the neck, thorax, and left lower limb, along with F-18 fluorodeoxyglucose (FDG)-avid lymphadenopathy in those regions (Figure 4). With no evidence of malignancy or inherited thrombophilia, a multidisciplinary discussion concluded that the findings were most consistent with septic thrombophlebitis involving the internal jugular vein, brachiocephalic vein, and SVC, with evidence of septic pulmonary emboli, a presentation consistent with Lemierre's syndrome.

An MRI of the neck performed on day 16 showed that the prevertebral collection had resolved. Based on infectious disease recommendations, the patient was continued on IV piperacillin-tazobactam for three weeks, followed by oral antibiotics. A cardiac workup was also pursued. Transthoracic echocardiogram on day 8 showed moderate global left ventricular (LV) hypokinesis and a positive agitated saline contrast study. Cardiac MRI revealed mild left ventricular dysfunction (ejection fraction (EF): 48%) with increased end-systolic volume. A transesophageal echocardiogram later identified a large thrombus in the SVC extending into the right atrium, but no evidence of endocarditis.

**Figure 5:**
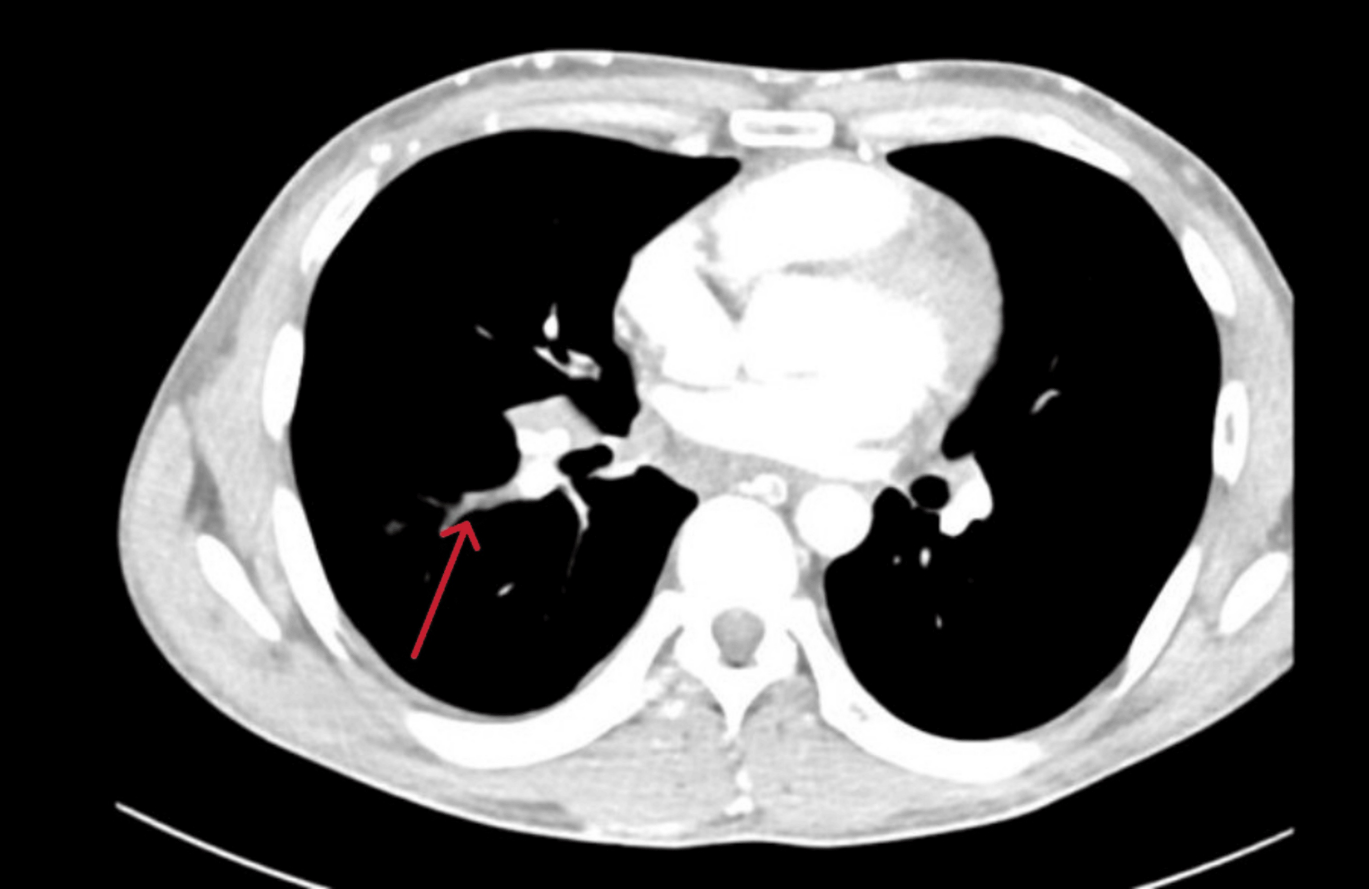
Computed tomography pulmonary angiography showing right-sided pulmonary emboli (red arrow)

Despite appropriate treatment, the patient had intermittent low-grade fevers. He had initially received a seven-day course of amoxicillin/clavulanate in the community for presumed pharyngitis before presenting to our facility. All blood cultures throughout admission remained negative. On day 20 of hospitalization, he developed hypotension (blood pressure (BP): 86/65 mmHg). A pulmonary CT angiogram revealed pulmonary emboli in the segmental and subsegmental branches of the right lower lobe, along with a 13 mm cavitary lesion consistent with a septic embolus (Figure 5). Anticoagulation was continued with enoxaparin 70 mg twice daily and later adjusted to 60 mg based on anti-Xa levels. On day 30, he was transitioned to oral rivaroxaban (15 mg twice daily for 14 days, then 20 mg daily).

Although thrombolysis and thrombectomy were discussed with the patient, he declined both, as well as bronchoalveolar lavage for microbiological testing. He was discharged on oral clindamycin 450 mg three times daily to complete a six-week antibiotic course and rivaroxaban as planned. He was referred for close outpatient follow-up with infectious disease, hematology, and pulmonology.

## Discussion

LS is a rare but serious complication of oropharyngeal infections, most commonly involving septic thrombophlebitis of the internal jugular vein (IJV). The infection typically spreads from the oropharynx to the parapharyngeal space via lymphatic or hematogenous routes due to the close anatomical relationship between these structures [2]. Thrombus formation often begins in the peritonsillar veins and extends into the IJV, leading to systemic complications such as septicemia, abscess formation, and pulmonary septic emboli [3]. The syndrome is most frequently caused by *Fusobacterium necrophorum*, a Gram-negative anaerobic bacterium found in the gastrointestinal tract, which accounts for 10% of acute sore throat cases and a significantly higher proportion of recurrent infections [4]. Other organisms, including *Fusobacterium nucleatum*, *F. gonidiaformans*, and *Streptococcus* species, may also be involved [5,6].

LS primarily affects young individuals, with 90% of cases occurring between the ages of 10 and 35, and shows a 2:1 male predominance [6,7]. Although its incidence has significantly declined due to widespread antibiotic use, rising antimicrobial resistance and variability in clinical presentation continue to pose diagnostic challenges. The diagnostic triad includes the following: (1) a primary infection in the head or neck region or evidence of septic metastases, (2) thrombosis of the IJV or adjacent veins, and (3) isolation of *F. necrophorum* from blood or sterile body fluids [8]. When not all criteria are fulfilled, the condition is termed "atypical LS." Our patient presented during the second phase of the disease, with signs of IJV and SVC thrombosis, without a clear history of oropharyngeal infection or positive cultures, highlighting the variability of clinical presentations and diagnostic limitations due to difficulty in culturing anaerobes.

In our case, diagnosis was heavily reliant on imaging findings. CT of the neck and thorax demonstrated thrombosis of the internal jugular vein, brachiocephalic vein, and superior vena cava, with associated prevertebral soft tissue edema and pulmonary septic emboli. These findings are consistent with reported imaging patterns in LS, where CT is preferred due to its sensitivity for detecting vessel wall inflammation, thrombosis, and metastatic septic foci [6,9]. However, in light of his markedly poor dentition, history of oropharyngeal symptoms, radiological evidence of IJV and SVC thrombosis, and pulmonary septic emboli, LS was strongly suspected despite the absence of a confirmed *Fusobacterium* infection. The patient's refusal to undergo invasive diagnostic procedures limited tissue-based confirmation of other potential diagnoses, such as lymphoma. Nevertheless, the combination of radiological and clinical features supported the diagnosis of atypical LS.

An unusual aspect of this case was the presence of widespread thrombosis, including in the lower extremities. This has not been commonly reported in LS and may represent a manifestation of sepsis-induced hypercoagulability. Workup for underlying thrombophilia, malignancy, and autoimmune disease was unrevealing, although lymphoma could not be ruled out due to the patient's refusal of lymph node biopsy [10]. The thrombus was also found to extend into the right atrium, raising the risk of further embolic events. While surgical thrombectomy was considered, the patient opted for medical management with anticoagulation, which is supported by evidence suggesting similar outcomes compared to surgery in cases of right atrial thrombus [11]. Pulmonary complications are common in LS, frequently presenting as necrotic cavitary lesions from septic emboli, along with pleural effusions and lung abscesses [1]. Hematogenous spread can also affect large joints and other organs, causing abscesses and rare vascular or central nervous system (CNS) complications. Central nervous system involvement, although unusual, may include meningitis, brain abscesses, and cerebral venous thrombosis [6,12].

Recent systematic analyses indicate that, although LS is classically characterized by venous thrombosis and septic emboli, cardiac involvement occurs in a notable subset of patients, with pericardial complications, infective endocarditis, and cardiac thrombi reported in up to 29% of cases with arterial involvement [12]. This cardiac involvement is associated with a higher risk of mortality (12% versus 3.3%) and long-term clinical sequelae (35% versus 9%) compared to patients without arterial or cardiac complications, suggesting that heart involvement may serve as a marker of disease severity [12].

Management involved the use of prolonged broad-spectrum antibiotics and anticoagulation, guided by clinical suspicion and radiological findings rather than culture results. Although a 2020 meta-analysis found no significant impact of anticoagulation on mortality or recanalization rates in LS, it remains a reasonable option, particularly in extensive or intracardiac thrombus [13]. Our patient improved with a combination of piperacillin-tazobactam and low-molecular-weight heparin, and later transitioned to rivaroxaban. Similar successful outcomes have been reported in patients treated based solely on clinical and imaging criteria. One such case involved a 14-year-old female patient who recovered fully with antibiotics and anticoagulation despite persistently negative cultures, reinforcing the importance of clinical judgment in atypical presentations [14].

## Conclusions

LS, although uncommon, should be promptly considered in young, otherwise healthy patients presenting with suggestive symptoms to prevent diagnostic delays. Imaging plays a crucial role in guiding both diagnosis and management, and careful clinical reassessment can help identify cases that might otherwise be missed. Continued follow-up is essential for monitoring complications and ensuring effective treatment outcomes.
